# Cerebral Malaria in a Patient with HIV, Hepatitis B, and Hepatitis C

**DOI:** 10.7759/cureus.2569

**Published:** 2018-05-02

**Authors:** Syed Mohammad Mazhar Uddin, Aatera Haq, Zara Haq, Uzair Yaqoob, Haider Shah, Syed Faheem Ali Kazmi

**Affiliations:** 1 Medicine, Civil Hospital Karachi, Karachi, Karachi, PAK; 2 Medicine, Civil Hospital Karachi, Karachi, PAK; 3 Sindh Medical College, Dow University of Health Sciences, Karachi, PAK

**Keywords:** malaria, hiv, hepatitis

## Abstract

Cerebral malaria is one of the most common causes of non-traumatic encephalopathy. A 25-year-old man who is a known intravenous and oral drug abuser presented to our clinic with fever and sore throat for two days prior and an altered level of consciousness for one day. On examination, the patient was icteric, and his Glasgow coma scale score on arrival was 10/15; he had dilated pupils reactive to light and a positive corneal reflex. All cranial nerves were intact; however, signs of meningeal irritation were positive. Motor examination showed an increased tone and rigidity in all limbs, patellar reflex was 3+, plantars were down-going, and clonus was negative. A fundoscopic examination was unremarkable. Additional investigations revealed he was positive for Plasmodium falciparum, HIV, hepatitis B, and hepatitis C. In addition, a test of his cerebrospinal fluid revealed evidence of cerebral malaria. We initiated artemether 120 mg, intravenous ceftriaxone 2 g, and 5% dextrose saline for the intermittent hypoglycemia. The patient’s condition eventually improved drastically. This case outlines the possible exacerbating effect of HIV on malaria, and it calls for HIV screening and staging alongside suspected malaria. This case also underlines the need for further evaluation of a potential protective role of hepatitis B and C to find an alternative therapeutic cure for malaria.

## Introduction

Malaria is an important parasitic disease, and its neurological complication, cerebral malaria, is one of the most common causes of non-traumatic encephalopathies in the world [1]. One study reported that 20% to 50% of the deaths in *Plasmodium falciparum* malaria cases were due to the involvement of the central nervous system [2]. We report a unique case of cerebral malaria from *P. falciparum* in a patient with HIV, hepatitis B, and hepatitis C, and we explore the potential influence of those concomitant infections on the patient’s cerebral malaria.

## Case presentation

A 25-year-old man, with no known comorbidities but with a known history of intravenous drug abuse (heroin) from the age of 18, presented with a concern of fever and sore throat lasting two days and an altered level of consciousness  lasting one day. According to the patient’s attendant, he was in his usual state of health two days prior when he developed a high-grade fever associated with a sore throat. Furthermore, patient’s attendant mentioned that he was found in an altered state of consciousness along with fecal and urinary incontinence. The patient had no history of trauma to the head as well as no positive history of seizures. There was no significant past medical or surgical history. The patient’s addiction history was positive for heroin, Hashish, and methamphetamine both orally as well as intravenously. On examination, his vital signs on arrival were as follows: blood pressure, 110/60 mmHg (reference, 120/80 mmHg); pulse, 100 bpm (reference, 70 to 100 bpm); respiratory rate, 30 breaths/minute (reference range, 18 to 22); and temperature, 102°F (reference, 98.6°F). Furthermore, in his general physical exam, he was positive for jaundice and had marks of cuts on his forearm, suggesting the possibility of multiple needle insertions. His abdominal, respiratory, and cardiovascular exam results were unremarkable. His Glasgow coma scale (GCS) on arrival was 10/15. He had dilated  pupils reactive to light, as well as a positive corneal reflex. All cranial nerves were intact; however, signs of meningeal irritation were present. A motor examination showed an increased tone in all limbs, positive rigidity in all four limbs,  his  patellar reflex was 3+, his plantars were down-going, and clonus (ankle and knee) was negative. Furthermore, the results of the fundoscopic examination were unremarkable.

We immediately did clinical laboratory testing of this patient. His laboratory test results are presented in Table 1. Given our region, we also tested for malaria and found the patient was positive for *Plasmodium falciparum*. Given the patient’s  intravenous drug abuse, we also tested his viral markers and HIV status, the results of which were positive for both hepatitis B and C and HIV, with a CD4 count of 270 (reference range, 500 to 1500). Immediately, the patient was catheterized with monitoring of urine output, and a nasogastric tube was passed. The patient was started on artemether,  120 mg, delivered in six doses spaced 12 hours apart, and intravenous ceftriaxone 2 g/day for five days. The patient had low random blood glucose levels of 60 mg/dL (reference range, 79 to 160 mg/dL), for which he was started on 5% dextrose saline intravenously as well. At this point, we were suspicious of many differentials such as cerebral malaria, bacterial/tuberculous/fungal meningitis, cytomegalovirus/herpes simplex virus encephalitis, drug overdose,  central nervous system (CNS) lymphoma, as well as toxoplasmosis. To arrive at a final diagnosis, we conducted a series of additional investigations. We ordered a chest X-ray and ultrasound of the abdomen, the findings of which were  unremarkable. To rule out meningitis, we did a lumbar puncture, and his cerebrospinal fluid (CSF) findings are presented  in Table 2. At the time of the lumbar puncture, his random blood glucose level was 102 mg/dL (reference range, 79  to 160 mg/dL). Results of the tests were negative for Cryptococcus antigen and tuberculosis. A computed tomography (CT)  scan and magnetic resonance image (MRI) of the brain were unremarkable, thereby ruling out ring-enhancing lesions due to tuberculoma, CNS lymphoma or toxoplasmosis. To rule out infective endocarditis, we tested blood cultures and conducted an echocardiographic exam, the results of which were both unremarkable. We also ordered a urine toxicology test given the patient’s status as a drug addict. The urine toxicology results were negative for any drug intoxication. By this point in our investigation, we became firm in our belief that cerebral malaria was the correct diagnosis.

**Table 1:** Patient baseline laboratory values. SGPT: Serum glutamic pyruvic transaminase; ESR: Erythrocyte sedimentation rate; CRP: C-reactive protein.

Analyte	Patient Level	Reference Range
Hemoglobin	12.8 g/dL	13.8–17.2 g/dL
Total leukocyte count	7.7 × 10^9^/L	4.5–11.0 × 10^9^/L
Platelet count	174 × 10^9^/L	150–400 × 10^9^/L
Blood urea nitrogen	17 mg/dL	7–20 mg/dL
Creatinine	0.9 mg/dL	0.6–1.2 mg/dL
Sodium	132 mEq/L	135–145 mEq/L
Potassium	7.1 mEq/L	3.5–5.5 mEq/L
Chloride	95 mEq/L	97–107 mEq/L
Total bilirubin	2.06 mg/dL	0.1–1.2 mg/dL
SGPT	175 units	7–56 units/L
ESR	87 mm/hr	0–22 mm/hr
CRP	260.3 mg/dL	<3 mg/dL

**Table 2:** Lumbar puncture findings. CSF: Cerebrospinal fluid; WBC: White blood cells.

Analyte	Patient Level	Reference Range
CSF glucose	48 mg/dL	45–80 mg/dL
CSF chloride	127 mEq/L	110–125 mEq/L
CSF protein	135 mg/dL	15–45 mg/dL
CSF WBC	0.019 cells/µL	0–5 cells/µL
CSF polymorphs	20	None
CSF lymphocyte	80 × 10^6^/L	<5 × 10^6^/L

However, after 10 days of treatment, the patient showed considerable improvement despite being very critical in condition. He was no longer in an altered level of consciousness, and his fever had subsided. Furthermore, his GCS improved to 15/15, and his neurological examination results were normal. His laboratory findings had also improved. The patient was discharged and referred to an HIV clinic and a hepatitis outpatient department for his viral infections; he was also referred to a rehabilitation program for his addiction.

## Discussion

Cerebral malaria is defined as a diffuse encephalopathy with unusual focal neurologic signs. Clinically, the patient is febrile, in an altered level of consciousness with a divergent gaze and a variable tone. There are signs of meningism, but to a lesser degree than meningitis [1]. Furthermore, as there are signs of multi-organ dysfunction, so the assessment of anemia, jaundice, and acidotic breathing is important for the overall prognosis of the patient. Rarely, the patient can also have disseminated intravascular coagulation, along with bleeding from the upper gastrointestinal tract as well as the skin. Furthermore, hypoglycemia is a common finding in about 8% of adults and 20% of children, and it usually does not occur with other signs of hypoglycemia [1]. Hence, frequent monitoring of blood glucose levels is important. On examination, direct ophthalmoscopy shows retinal hemorrhages in 15% of cases, and indirect ophthalmoscopy also reveals hemorrhages in a much higher percentage of the population [1]. The pupillary reactions are usually normal, corneal reflexes are present (except in deep coma), and the range of eye movements are full. However, rarely, palsy of the third cranial nerve can occur, and the gaze is usually dysconjugate. A motor exam usually reveals a variable tone, absent abdominal reflexes, up-going planters, and, rarely, patellar and ankle clonus can be elicited [1]. Furthermore, studies also report that at least one episode of seizures can occur in cases of cerebral malaria, with status epilepticus being uncommon [1,3].

Our patient was also febrile, with an altered level of consciousness and positive signs of meningism. Furthermore, hypoglycemia was also present intermittently and treated via dextrose saline administration accordingly. Our patient also had a dysconjugate gaze, with normal pupillary reflexes and corneal reflexes with a full range of eye movement. However, his fundoscopic examination was normal, and there were no episodes of seizures. In his motor exam, the planters were down going, and we could not appreciate clonus (patellar and ankle). The findings of CSF analyses in cases of cerebral malaria usually reveal characteristically elevated lactate levels, mild pleocytosis (10 to 50 cells/mm^3^), as well as mild elevations of proteins (up to 200 mg/dL). CSF glucose levels can be low, but this should be checked cautiously as, in cerebral malaria, plasma glucose levels are already on the lower side. Hence, studies have suggested the use of the ratio of CSF glucose to serum glucose for future studies. In our patient, CSF lactate levels could not be assessed, but there was mild pleocytosis and a marked increase in protein levels; glucose levels were normal. This marked increase of proteins and mild pleocytosis can also help differentiate viral encephalitis and bacterial meningitis, respectively [4,5]. Furthermore, CT scans and MRI in patients of cerebral malaria are usually normal or show edema and cortical or subcortical infarcts in the watershed zone in about 15% to 20% of patients [5]. Our patient had an unremarkable CT scan and MRI.

The effects of HIV, hepatitis B, and hepatitis C on cerebral malaria are scarcely documented. To our knowledge, this is the only case to document cerebral malaria with all three viral infections at the same time. Literature reveals that in HIV, the inflammatory immune response in the brain and destruction of the blood-brain barrier causes an increase in the susceptibility and severity of cerebral malaria. Additionally, the poor response to pathogens impairs the body’s ability to remove the malarial infection, thereby increasing the parasitemia and leading to severe anemia [6]. However, for hepatitis B, studies suggest a difference in opinion, with one study stating that a hepatitis B infection may also exacerbate​​​​​ *falciparum *malaria, thereby increasing the risk of morbidity and mortality [7], whilst others state it either has no significant effects on cerebral malaria or can have some protective effects [8,9]. As for hepatitis C, a few authors suggest it slows down the emergence of *P. falciparum*, indicating a potential protective effect [10].

## Conclusions

Overall, the epidemiology of all the co-infections with cerebral malaria has not been profoundly studied, and our case report will raise awareness of this phenomenon. Malaria and HIV should be considered significant public health issues worldwide, with special emphasis on the Karachi, Pakistan region. In addition, future studies involving patients with cerebral malaria should be screened and staged for HIV for more valuable insight about its effect on cerebral malaria. Additional studies are needed for hepatitis B and hepatitis C to determine their effects on cerebral malaria as the data are scarce in the literature. Moreover, if either hepatitis B or C do confer a protective effect, this protective effect should be explored to determine a potential alternative therapeutic remedy for malaria.
